# Supplementary material for: Cas9-AAV6 gene correction of beta-globin in autologous HSCs improves sickle cell disease erythropoiesis in mice
Source: Nat Commun. 2021 Jan 29;12:686. doi: 10.1038/s41467-021-20909-x (PMC7846836; doi:10.1038/s41467-021-20909-x)
Supplement: Supplementary file 1 — Supplementary Information [file 41467_2021_20909_MOESM1_ESM.pdf]

SUPPLEMENTARY INFORMATION

Cas9-AAV6 gene correction of beta-globin in autologous HSCs improves sickle cell disease erythropoiesis in mice

Wilkinson and Dever et al.

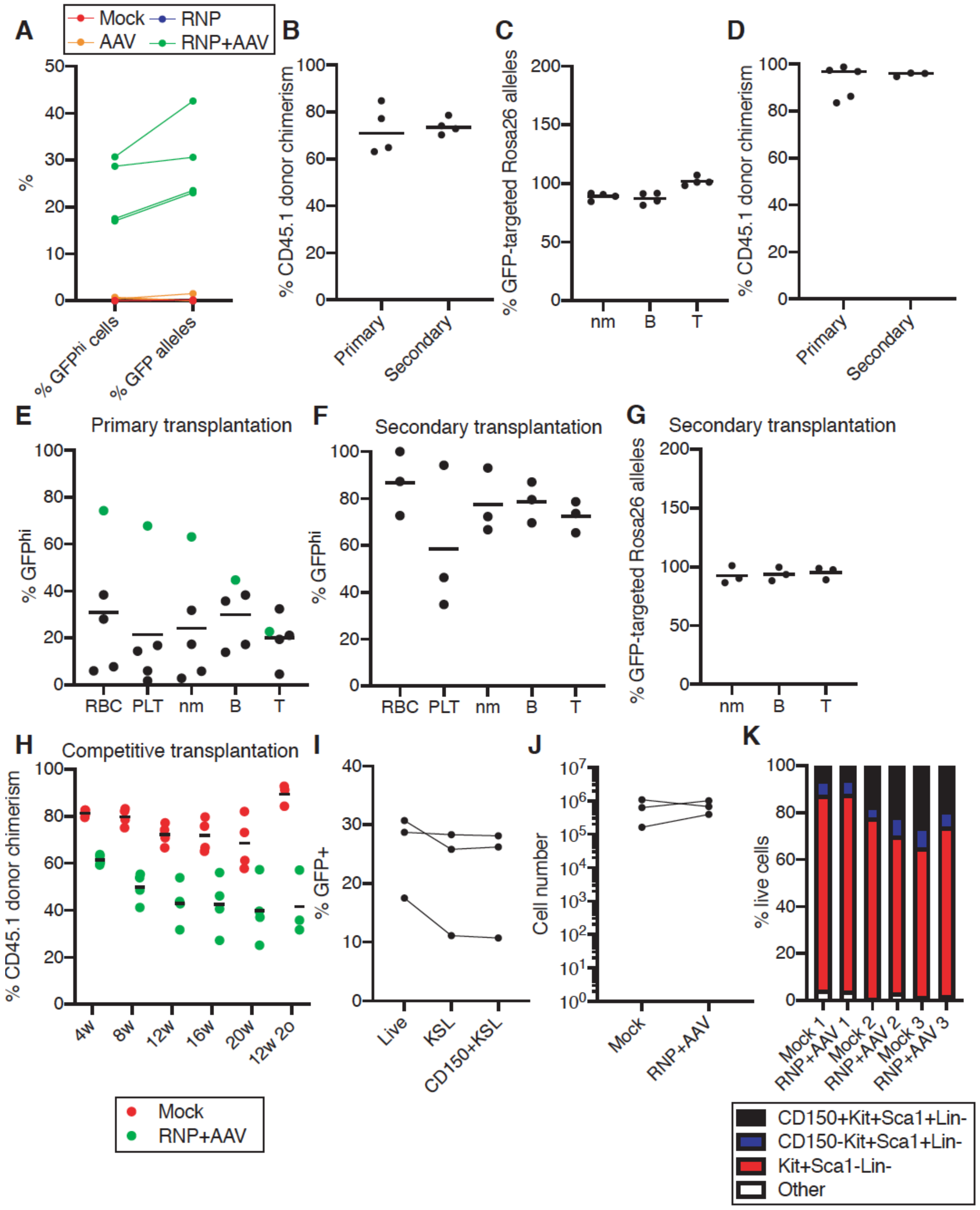

Supplementary Figure 1: Evaluation of *Rosa26* gene targeting in mouse HSCs

(**A**) Frequency of GFPhi cells (measured by flow cytometry) and *Rosa26*-targeted GFP alleles (measured by ddPCR) in the day-14 HSPC cultures, for mock (n=3 independent cell cultures),

AAV-only (n=3 independent cell cultures), RNP-only (n=3 independent cell cultures), and RNP+AAV gene-edited (n=4 independent cell cultures) samples.

(**B**) Mean percentage donor CD45.1 chimerism at in transplant recipients described in **Figure 1E-F** (n=4 mice).

(**C**) Mean frequency of genomic *GFP* targeted *Rosa26* alleles in GFP^{hi} PB lineages collected from secondary recipients at 12-weeks in **Figure 1F**, measured by ddPCR (n=4 mice).

(**D**) Mean percentage donor CD45.1 chimerism for a biological replicate of the transplant described in **Figures 1E-F.** Day 14 cultures (derived from 200 HSCs; ~$2x10^5$ cultured cells) transplanted alongside $2x10^5$ helper whole bone marrow cells (WBMCs) per recipient (n=5 mice). After 20-weeks, secondary transplantation was performed (n=3 mice) using $1x10^6$ WBMCs from mouse 1.

(**E**) Mean frequency of GFP^{hi} cells in PB lineages of primary recipients at 20-weeks post-transplantation from **Supplementary Figure 1D**, measured by flow cytometry (n=5 mice).

(**F**) Mean frequency of GFP^{hi} cells in PB lineages of secondary recipients at 20-weeks post-transplantation from **Supplementary Figure 1D**, measured by flow cytometry (n=3 mice).

(**G**) Mean frequency of genomic *GFP* targeted *Rosa26* alleles in GFP^+ PB lineages collected from secondary recipients in **Supplementary Figure 1F**, measured by ddPCR (n=3 mice).

(**H**) Mean frequency of $CD45.1^+$ donor cells in a competitive transplantation assay of day 14 HSPC cultures (derived from 200 HSCs; ~$2x10^5$ cultured cells) competed against $1x10^6$ WBMCs. HSPC cultures either mock electroporated or *Rosa26*-edited (RNP+AAV) at day 7. Mean chimerism at 4-20-weeks post-transplantation in primary recipients (n=4 mice) and 12-weeks post-transplantation in secondary recipients (n=3 mice; $1x10^6$ WBMCs from all primary recipients used for secondary transplantation).

(**I**) Frequency of GFP^{hi} cells in phenotypic cell types of gene edited day-14 HSC cultures (n=3 independent cell cultures).

(**J**) Cell number of day-14 HSPC cultures after day-7 mock or RNP+AAV gene editing. HSPC cultures derived from 1000 $CD150^+CD34^-Kit^+Sca1^+Lin^-$ BM cells (n=3 independent cell cultures).

(**K**) Frequency of phenotypic cell types within 3 mock and 3 RNP+AAV *Rosa26*-edited HSPC cultures at day 14.

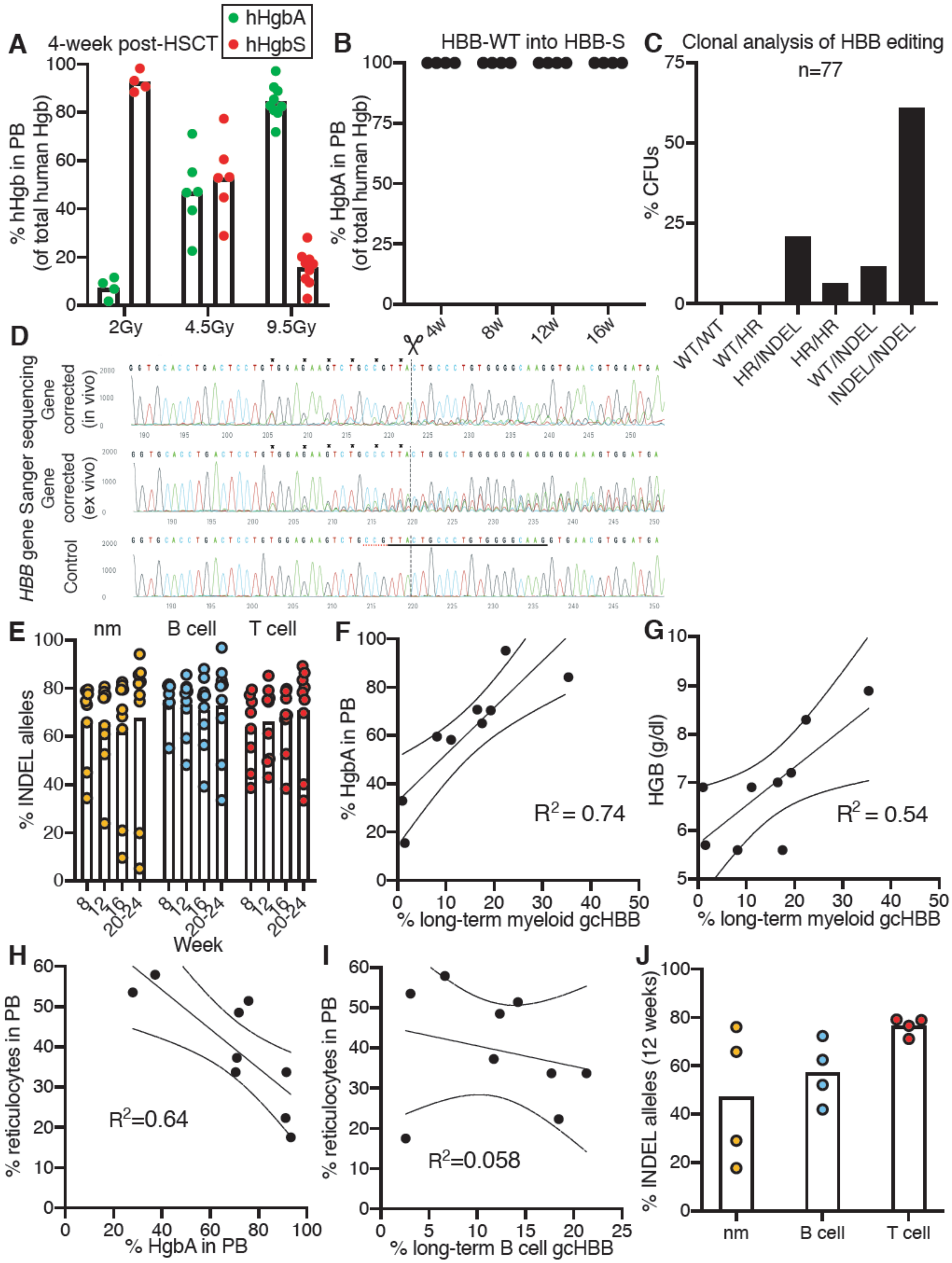

Supplementary Figure 2: Evaluation of *HBB* gene correction in Townes-SCD mouse HSCs

(**A**) Mean percentage of human Hemoglobin S (hHgbS) and human Hemoglobin A (hHgbA) tetramers in Townes-SCD *HbS* recipients, 4-weeks after transplantation with gene-corrected day

14 HSPCs described in **Figure 2A** (n=4 mice for 2 Gy; n=6 mice for 4.5 Gy; n=9 mice for 9.5 Gy). Recipient mice given 4 Gy and 9.5 Gy radiation doses received blood transfusions to support survival.

(**B**) Mean percentage of hHgbA in Townes-SCD *HbS* recipient mice following transplantation of day 14 HSPC cultures derived from 500 *HBB-WT* HSCs (n=4 mice).

(**C**) Frequency of *HBB* gene editing events in colony forming units generated from *HBB*-gene edited Townes-SCD HSPCs (n=77 colonies collected from 3 independent gene editing experiments).

(**D**) Sanger sequencing traces for the genomic target of the *HBB* gene correction strategy, amplified from control and gene-corrected in vitro HSPC cultures (derived from Townes SCD HSCs), and myeloid (nm) cells from a recipient mouse 16-week post-transplantation.

(**E**) Mean frequency of INDEL *HBB* alleles in the primary Townes-SCD recipients described in **Figure 2C** (n=9 mice).

(**F**) X-Y linear correlations between percentage hHgbA and percentage myeloid (nm) *HBB* allelic correction at 16-weeks post-transplantation (n=9 mice).

(**G**) X-Y linear correlations between total PB HGB levels and percentage myeloid (nm) *HBB* allelic correction at 16-weeks post-transplantation (n=9 mice).

(**H**) X-Y linear correlations between PB reticulocyte frequency and percentage hHgbA at 16-weeks post-transplantation (n=9 mice).

(**I**) X-Y linear correlations between PB reticulocyte frequency and B cell gene correction at 16-weeks post-transplantation (n=9 mice).

(**J**) Mean frequency of INDEL *HBB* alleles in the secondary recipients at 12-weeks post-transplantation for mice described in **Figure 2H** (n=4 mice).

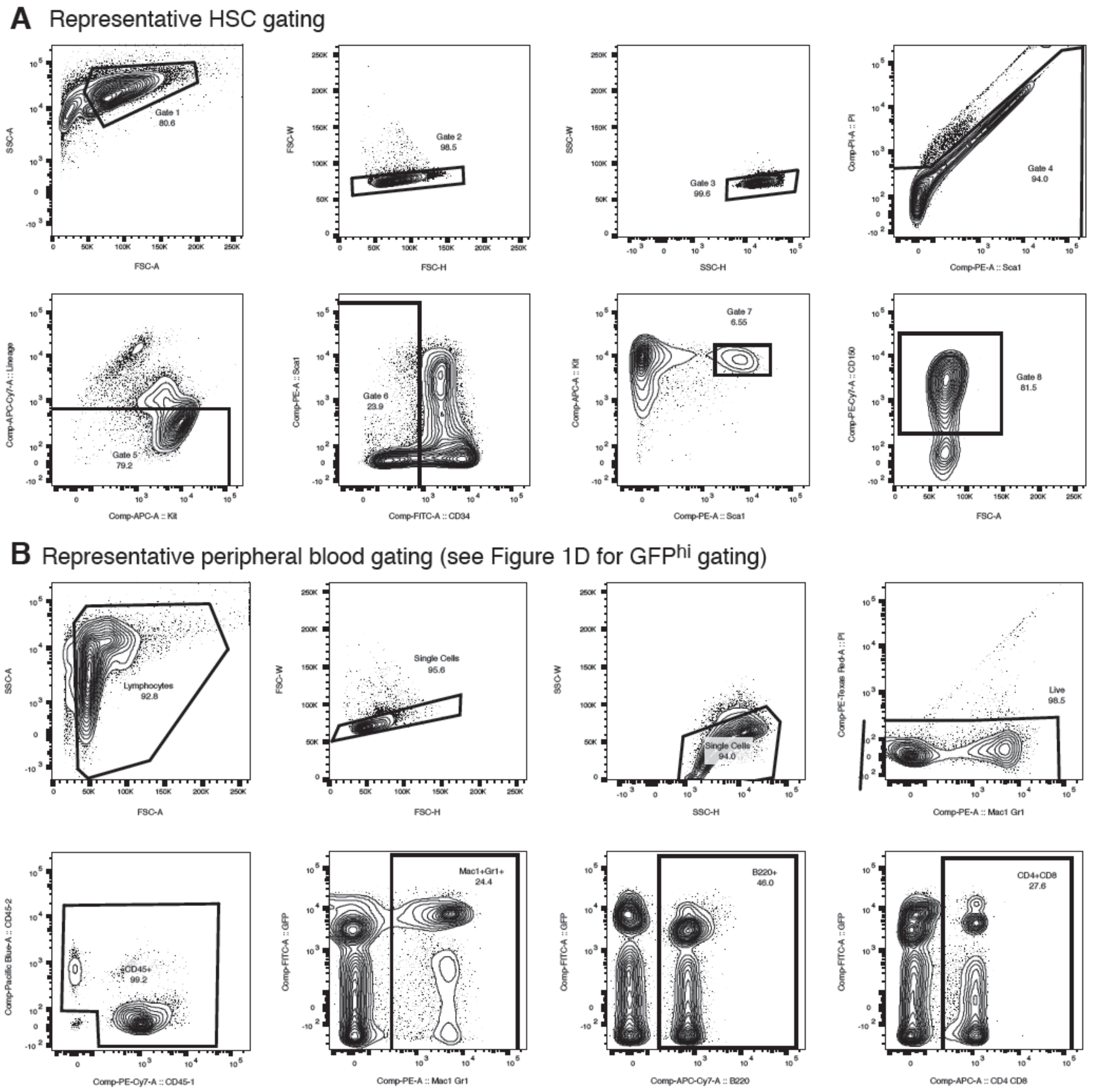

Supplementary Figure 3: Representative FACS gating

(**A**) Representative gating for sorting adult mouse BM CD150^{+}CD34^{-}Kit^{+}Sca1^{+}Lineage^{-} HSCs.

(**B**) Representative gating for sorting mouse peripheral blood leukocyte cell populations. Representative GFP^{hi} gating displayed in **Figure 1D**.

Supplementary Table 1: Antibodies

Antibody	Dilution	Source	Identifier
Biotin anti-CD4	1:2800	eBioscience	Cat#13-0041-85
Biotin anti-CD8	1:2800	eBioscience	Cat# 13-0081-86
Biotin anti-CD45R/B220	1:1400	eBioscience	Cat# 36-0452-85
Biotin anti-TER-119	1:700	eBioscience	Cat# 13-5921-85
Biotin anti-Ly-6G/Ly-6C (RB6-8C5)	1:700	eBioscience	Cat# 13-5931-85
Biotin anti-CD127 (A7R34)	1:1400	eBioscience	Cat# 13-1271-85
APC anti-c-Kit (2B8)	1:100	eBioscience	Cat# 17-1171-83
FITC anti-CD34 (RAM34)	1:100	eBioscience	Cat# 11-0341-85
PE-Cy7 anti-CD150 (TC15-12F12.2)	1:350	BioLegend	Cat# 115914
PE anti-Ly-6A/E (Sca-1) (D7)	1:700	BioLegend	Cat# 122508
Streptavidin-APC/eFluor780	1:700	eBioscience	Cat# 47-4317-82
PE-Cy7 anti-CD45.1	1:500	BioLegend	Cat# 110730
BrilliantViolet421 anti-CD45.2 (104)	1:400	BioLegend	Cat# 109832
PE anti-Ly-6G/Ly-6C (RB6-8C5)	1:2000	eBioscience	Cat# 12-5931-82
PE anti-CD11b (M1/70)	1:2000	eBioscience	Cat# 12-0112-82
APC-eFluor780 anti CD45R (RA3-6B2)	1:1000	eBioscience	Cat# 47-0452-82
APC anti-CD4 (RM4-5)	1:2000	eBioscience	Cat# 17-0042-83
APC anti-CD8 (53-6.7)	1:2000	eBioscience	Cat# 17-0081-83
APC-eFluor780 anti-Ter119 (TER119)	1:50	eBioscience	Cat# 47-5921-82
APC anti-CD42 (1C2)	1:50	Biolegend	Cat# 148506
Pacific Blue anti-CD41 (MWReg30)	1:50	Biolegend	Cat# 133936
Streptavidin-PE	1:50	eBiosciences	Cat# 12-4317-87

Supplementary Table 2: Synthetic sgRNAs

Target	Sequence
Rosa26[18,19]	actccagtctttctagaaga
HBB[3]	cttgccccacagggcagtaa

Supplementary Table 3: Cloning primer sequences

Primer name	Primer sequence
Rosa26 genomic region forward	ccgacgtctcgtcgctgattg
Rosa26 genomic region reverse	ccctggactgagaataggccc
Rosa26 LHA forward Gibson	agcgagcgcgcagagagggagtggccaactccatcactaggggttcctgc ggccgcatccgccggccagc
Rosa26 LHA reverse Gibson	ggaggcctagggataacagggtaattctagaaagactggagttgcagatcac
Rosa26 RHA forward Gibson	tgaggcggaaagaacgtttcgcgccagatgggcgggagtctt
Rosa26 RHA reverse Gibson	agcgagcgcgcagagagggagtggccaactccatcactaggggttcc tgcggccgcgaaaatgccaatgctctgtctaggg
Ubc-GFP-pA forward Gibson	gatctgcaactccagtctttctagaattaccctgttatccctaggcct
Ubc-GFP-pA reverse Gibson	ctgcccagaagactcccgcccatctggcgcgaaacgttctttcc

Supplementary Table 4: Genotyping primer/probe sequences

Primer name	Primer sequence
HBB out forward	aggaagcagaactctgcacttca
HBB in reverse	agtcagtgcctatcagaaacccaagag
HBB ddPCR forward	tcactagcaacctcaaacagac
HBB ddPCR reverse	cctgtcttgtaaccttgatacc
HR probe (HEX)	tgactcctgaggaAaaAtcCgcAgtCa
Reference probe (HEX)	acgtggatgaagttggtggtgagg
WT probe (FAM)	ccccacagggcagtaacggcagacttc
Rosa26-GFP in-out forward	aagggggaggattgggaaga
Rosa26-GFP in-out reverse	acagcctcgatttgtggtgt
Rosa26-GFP in-out probe (FAM)	catgctggggatgcggtggg
Genomic reference forward	aggcttggcactaaatgggt
Genomic reference reverse	gtccaacggctcagcctgca
Genomic reference probe (HEX)	gaaaggacctccaagccgtt
