# Supplementary material for: Cas9-AAV6 gene correction of beta-globin in autologous HSCs improves sickle cell disease erythropoiesis in mice
Source: Nat Commun. 2021 Jan 29;12:686. doi: 10.1038/s41467-021-20909-x (PMC7846836; doi:10.1038/s41467-021-20909-x)
Supplement: Supplementary file 3 — Reporting Summary [file 41467_2021_20909_MOESM3_ESM.pdf]

nature research

Corresponding author(s): Matthew H. Porteus, Hiromitsu Nakauchi, Adam C. Wilkinson

Last updated by author(s): Nov 11, 2020

Reporting Summary

Nature Research wishes to improve the reproducibility of the work that we publish. This form provides structure for consistency and transparency in reporting. For further information on Nature Research policies, see our Editorial Policies and the Editorial Policy Checklist.

Statistics

For all statistical analyses, confirm that the following items are present in the figure legend, table legend, main text, or Methods section.

n/a	Confirmed	
☐	☒	The exact sample size (*n*) for each experimental group/condition, given as a discrete number and unit of measurement
☐	☒	A statement on whether measurements were taken from distinct samples or whether the same sample was measured repeatedly
☒	☐	The statistical test(s) used AND whether they are one- or two-sided<br>*Only common tests should be described solely by name; describe more complex techniques in the Methods section.*
☒	☐	A description of all covariates tested
☒	☐	A description of any assumptions or corrections, such as tests of normality and adjustment for multiple comparisons
☐	☒	A full description of the statistical parameters including central tendency (e.g. means) or other basic estimates (e.g. regression coefficient) AND variation (e.g. standard deviation) or associated estimates of uncertainty (e.g. confidence intervals)
☒	☐	For null hypothesis testing, the test statistic (e.g. *F*, *t*, *r*) with confidence intervals, effect sizes, degrees of freedom and *P* value noted<br>*Give P values as exact values whenever suitable.*
☒	☐	For Bayesian analysis, information on the choice of priors and Markov chain Monte Carlo settings
☒	☐	For hierarchical and complex designs, identification of the appropriate level for tests and full reporting of outcomes
☒	☐	Estimates of effect sizes (e.g. Cohen's *d*, Pearson's *r*), indicating how they were calculated

Our web collection on statistics for biologists contains articles on many of the points above.

Software and code

Policy information about availability of computer code

Data collection	BD FACS Diva 8, Biorad QuantaSoft 1.7
Data analysis	FlowJo 10, Prism 7, QuantaSoft 1.7

For manuscripts utilizing custom algorithms or software that are central to the research but not yet described in published literature, software must be made available to editors and reviewers. We strongly encourage code deposition in a community repository (e.g. GitHub). See the Nature Research guidelines for submitting code & software for further information.

Data

Policy information about availability of data

All manuscripts must include a data availability statement. This statement should provide the following information, where applicable:

- Accession codes, unique identifiers, or web links for publicly available datasets
- A list of figures that have associated raw data
- A description of any restrictions on data availability

All datasets available upon request.

Field-specific reporting

Life sciences study design

All studies must disclose on these points even when the disclosure is negative.

Sample size	No statistical methods were used to predetermine the experimental sample size. Sample sizes were determined based on mouse availability.
Data exclusions	No data were excluded.
Replication	For all figures, multiple independent experiments were performed and all attempts at replicating observation as described in the manuscript were successful. Number of biological replicates varied between experiments and are detailed within the figure legend text.
Randomization	Recipient mice were randomly selected for transplantation groups.
Blinding	Blinding was not performed.

Reporting for specific materials, systems and methods

We require information from authors about some types of materials, experimental systems and methods used in many studies. Here, indicate whether each material, system or method listed is relevant to your study. If you are not sure if a list item applies to your research, read the appropriate section before selecting a response.

Materials & experimental systems

n/a	Involved in the study	
☐	☒	Antibodies
☒	☐	Eukaryotic cell lines
☒	☐	Palaeontology and archaeology
☐	☒	Animals and other organisms
☒	☐	Human research participants
☒	☐	Clinical data
☒	☐	Dual use research of concern

Methods

n/a	Involved in the study	
☒	☐	ChIP-seq
☐	☒	Flow cytometry
☒	☐	MRI-based neuroimaging

Antibodies

Antibodies used

Biotin anti-mouse CD4 eBioscience Cat#13-0042-85 (1:2800)
Biotin anti-mouse CD8 eBioscience Cat# 13-0081-86 (1:2800)
Biotin anti-mouse CD45R/B220 eBioscience Cat# 13-0452-85 (1:1400)
Biotin anti-mouse TER-119 eBioscience Cat# 13-5921-85 (1:700)
Biotin anti-mouse Ly-6G/Ly-6C (RB6-8C5) eBioscience Cat# 13-5931-85 (1:700)
Biotin anti-mouse CD127 (A7R34) eBioscience Cat# 13-1271-85 (1:1400)
APC anti-mouse c-Kit (2B8) eBioscience Cat# 17-1171-83 (1:100)
FITC anti-mouse CD34 (RAM34) eBioscience Cat# 11-0341-85 (1:100)
PE-Cy7 anti-mouse CD150 (TC15-12F12.2) BioLegend Cat# 115914 (1:350)
PE anti-mouse-Ly-6A/E (Sca-1) (D7) BioLegend Cat# 122508 (1:700)
Streptavidin-APC/eFluor780 eBioscience Cat# 47-4317-82 (1:700)
PE-Cy7 anti-mouse CD45.1 BioLegend Cat# 110730 (1:500)
BrilliantViolet421 anti-mouse CD45.2 (104) BioLegend Cat# 109832 (1:400)
PE anti-mouse Ly-6G/Ly-6C (RB6-8C5) eBioscience Cat# 12-5931-82 (1:2000)
PE anti-mouse CD11b (M1/70) eBioscience Cat# 12-0112-82 (1:2000)
APC-eFluor780 anti-mouse CD45R (RA3-6B2) eBioscience Cat# 17-0452-83 (1:1000)
APC anti-mouse CD4 (RM4-5) BioLegend Cat# 100516 (1:2000)
APC anti-mouse CD8 (53-6.7) eBioscience Cat# 17-0081-83 (1:2000)
APC-eFluor780 anti-Ter119 (TER119) eBioscience Cat# 47-5921-82 (1:50)
Pacific Blue anti-CD41 (MWReg30) Biolegend Cat# 133936 (1:50)
APC anti-CD42 (1C2) Biolegend Cat# 148506 (1:50)
Streptavidin-PE eBiosciences Cat# 12-4317-87 (1:50)

Validation

All antibodies used in this study were validated by the manufacturer for mouse and for flow cytometry. Validation statements can be found on the manufacturer websites using the Cat# detailed above (www.biolegend.com; www.thermofisher.com/us/en/home/life-science/antibodies/ebioscience ; www.bdbiosciences.com).

Animals and other organisms

Policy information about studies involving animals; ARRIVE guidelines recommended for reporting animal research

Laboratory animals	All mice were 8-12 weeks when experiments were started. Male and female mice were used in this study.<br>C57BL/6 mice - Jackson Laboratory (000664)<br>PepBoyJ mice - Jackson Laboratory (002014)<br>B6;129. Homozygous for Hbbtm3(HBG1,HBB)Tow, Homozygous for Hbatm1(HBA)Tow - Jackson Laboratory (013071)<br>B6;129. Homozygous for Hbbtm2(HBG1,HBB*)Tow, Homozygous for Hbatm1(HBA)Tow - Jackson Laboratory (013071)
Wild animals	This study did not involve wild animals.
Field-collected samples	This study did not involve field-collected samples
Ethics oversight	All animal experiments were approved by the Administrative Panel on Laboratory Animal Care at Stanford University.

Note that full information on the approval of the study protocol must also be provided in the manuscript.

Flow Cytometry

Plots

Confirm that:

- [x] The axis labels state the marker and fluorochrome used (e.g. CD4-FITC).
- [x] The axis scales are clearly visible. Include numbers along axes only for bottom left plot of group (a 'group' is an analysis of identical markers).
- [x] All plots are contour plots with outliers or pseudocolor plots.
- [x] A numerical value for number of cells or percentage (with statistics) is provided.

Methodology

Sample preparation	Bone marrow, peripheral blood, and in vitro cell cultures were prepared into a single cell suspension in PBS. Bone marrow cells were cKit-enriched using an MACS LS column (Miltenyi). Red blood cell lysis using aqueous 140 mM ammonium chloride was performed on peripheral blood cells. Cells were filters (40uM) before FACS.
Instrument	BD FACS AriaII
Software	FACS Diva for data collection, FlowJo for data analysis.
Cell population abundance	FACS machine cell sorting efficiency confirmed by flow cytometric analysis of post-sorted cells where feasible.
Gating strategy	FSC-A/SSC-A for mononuclear cells, FSC-H/FSC-W followed by SSC-H/SSC-W for singlets, PI/Sca1 for PI- live cells, Lineage-cocktail/cKit for Lin- cells, Sca1/CD34 for CD34-/lo Lin-, cKit/Sca1 for CD34-/lo cKit+ Sca1+ Lin-, CD150/blank channel for CD150+ CD34-/lo Kit+ Sca1+ Lin-. For peripheral blood analysis, live cells were gated based on positive and negative markers.

- [x] Tick this box to confirm that a figure exemplifying the gating strategy is provided in the Supplementary Information.
